# Airway epithelia clear rhinovirus infection via two waves of cell extrusion

**DOI:** 10.1126/sciadv.aef2562

**Published:** 2026-09-25

**Authors:** Faith Fore, Dustin C. Bagley, Rocio Teresa Martinez-Nunez, Julia Aniscenko, Sebastian Johnston, Jody Rosenblatt

**Affiliations:** ^1^ The Randall Centre for Cell and Molecular Biophysics, School of Basic and Medical Biosciences, King’s College London, London, UK.; ^2^ The Francis Crick Institute, London, UK.; ^3^ Asthma UK Centre in Allergic Mechanisms of Asthma, London, UK.; ^4^ King’s College London, Department of Infectious Diseases, School of Immunology and Microbial Sciences, Faculty of Life Sciences and Medicine, King’s College London, Guy's Hospital, London, UK.; ^5^ National Heart and Lung Institute, Imperial College London, London, UK.; ^6^ School of Cancer and Pharmaceutical Sciences, King’s College London, London SE1 1UL, UK.

## Abstract

Epithelial barriers represent the first line of defense against pathogens, yet their role in innate immunity is typically relegated to pathogen detection and immune cell recruitment. This perspective ignores a fundamental biological principle: Epithelia defended against pathogens long before complex immune systems evolved. Here, we demonstrate that human bronchial epithelium retains this ancestral capacity, autonomously clearing rhinovirus (RV) within 24 hours by selectively extruding infected cells, a process that we term virus-induced cell extrusion (VICE). VICE occurs in two waves: a rapid response occurring independently of complete virus entry, followed by a replication-dependent wave. Barrier-defective epithelia that cannot extrude do not eliminate RV. While VICE maintains barrier integrity and reduces local infection, it also expels virus-laden cells, which can infect fresh epithelia. Thus, VICE enables leukocyte-independent epithelial defense while inadvertently promoting viral spread, redefining epithelia as central to viral pathogenesis and host protection.

## INTRODUCTION

Rhinoviruses (RVs), the leading cause of the common cold, are nonenveloped positive-sense single-stranded RNA viruses of the family Picornaviridae and genus *Enterovirus* (*1*–*3*). RV infections are typically mild but can cause severe respiratory disease in young children, older individuals, immunocompromised patients, and individuals with chronic lung diseases, including asthma, chronic obstructive pulmonary disease, and cystic fibrosis (*4*). RVs are classified into three species: RV-A, RV-B, and RV-C. RV-A and RV-B are further subdivided into major and minor groups based on receptor usage, with the major group binding intercellular adhesion molecule–1, whereas the minor group uses the low-density lipoprotein receptor, and RV-C binds cadherin-related family member 3 (*2*, *5*).

Human airway epithelia (HAE) form the first physical barrier encountered by inhaled pathogens and environmental insults. Beyond coordinating innate and adaptive immune responses, airway epithelia maintain tissue integrity through tightly regulated processes of cell turnover and repair. We previously discovered a highly conserved, primordial process that drives most epithelial cell death called cell extrusion (*6*–*12*). During normal homeostasis, physical crowding triggers live-cell extrusion through Piezo1 activation inducing contraction of a basolateral actin-myosin ring that seamlessly squeezes excess cells out apically to maintain optimal cell density (*7*–*11*). A Piezo1-independent extrusion pathway also removes apoptotic or damaged cells, thereby preventing barrier function defects (*6*, *9*, *10*).

Extrusion is not limited to removal of only dying, damaged, or supernumerary cells but is developing a growing role in innate immunity. Bacterial pathogens such as *Listeria monocytogenes*, *Salmonella enterica* serovar Typhimurium, and *Shigella flexneri* have all been linked to extrusion-associated epithelial responses (*13*–*18*). Rotavirus and enterovirus A71 can induce extrusion in intestinal epithelia and colonic organoids, respectively (*19*, *20*). Additionally, RV infection can induce shedding of ciliated airway epithelial cells (*21*–*24*), suggesting that extrusion may also play a role in RV clearance. However, other pathogens can hijack the extrusion pathway to potentially prevent their elimination, leading to different pathogenic outcomes (*25*). Thus, it is important to identify which pathogens activate extrusion, what pathways activate it, and how extrusion might affect infection clearance and spread. Here, we show that epithelia sheets alone can reduce RV infection using two waves of virus-induced cell extrusion (VICE). The first wave is rapid and independent of dynamin-dependent viral entry, relying on stretch-activated channels (SACs), whereas the second depends on viral replication and apoptosis.

## RESULTS

### Human RV infection leads to extrusion of infected epithelial cells

**Fig. 1.**
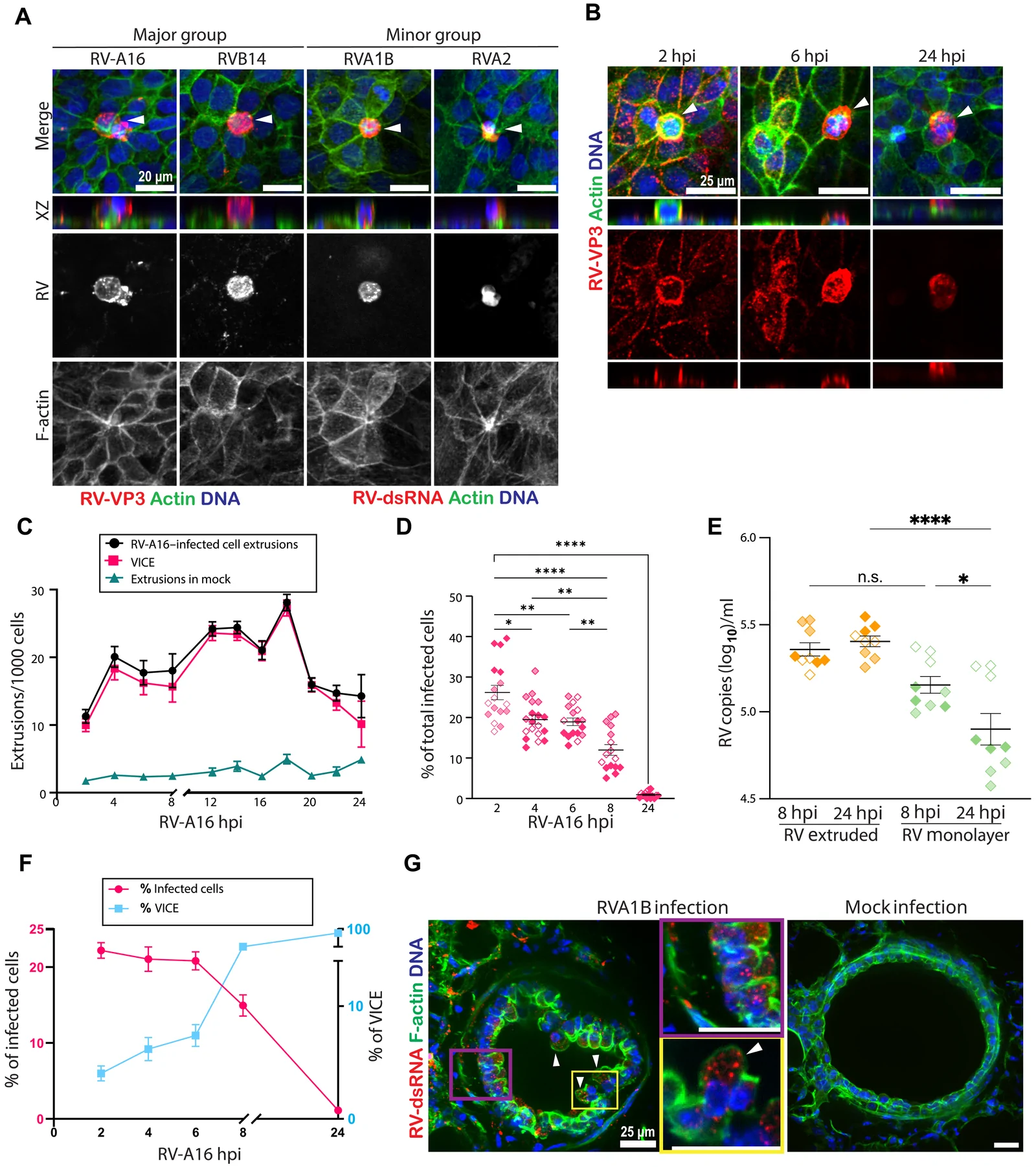
Human rhinovirus infection induces extrusion of infected epithelial cells. (**A**) Confocal projections of 16HBE14o^−^ monolayers infected for 24 hours with major-group (RV-A16 and RVB14) or minor-group (RVA1B and RVA2) rhinoviruses and stained for VP3 or dsRNA, respectively, F-actin and DAPI. (**B**) Time course of RV-A16 infection (MOI of 3) at 2, 6, and 24 hours postinfection (hpi), quantified in (C) and (D). (**C**) Quantification of total extrusion events, VICE events, and mock extrusion events over time following RV-A16 infection. VICE events were defined as extrusion events involving VP3^+^ cells. (**D**) Percentage of VP3^+^ cells remaining over time after RV-A16 infection. (**E**) RV RNA levels in extruded and monolayer-associated cells at 8 and 24 hpi, normalized to GAPDH and SDHA. (**F**) Relationship between the percentage of infected cells and the percentage of infected cells undergoing extrusion (VICE) over time following RV-A16 infection. % Infected cells is VP3^+^ cells/total cells, whereas % VICE is extruded VP3^+^ cells/total VP3^+^ cells. (**G**) Confocal projections of mouse lung slices infected with RVA1B for 24 hours versus mock controls, immunostained for dsRNA, F-actin, and DAPI. The purple-boxed region shows a higher magnification of the RVA1B-infected epithelium, highlighting dsRNA-positive epithelial cells. The yellow-boxed region highlights VICE. *n* ≥ 3 biological replicates, indicated by differentially shaded points, with mean and SEM. *P < 0.05, **P < 0.01, and ****P < 0.0001 from Welch’s ANOVA with Games-Howell post hoc test (D) and Mann-Whitney test (E). White arrowheads indicate extruding cells. n.s., not significant.

To investigate how intact epithelial monolayers respond to RV infection, we infected the simian virus 40–transformed human bronchial epithelial cell line (16HBE14o^−^) with RVA1B or RVA2 (minor group), RVB14 or RV-A16 (major group) at a multiplicity of infection (MOI) of 3 for 24 hours. RV-infected cells were identified by immunostaining for the capsid protein VP3 (major group) or double-stranded RNA (dsRNA) (minor group), actin with phalloidin, and nuclei with 4′,6-diamidino-2-phenylindole (DAPI). Confocal imaging revealed that both major and minor RV strains triggered extrusion of infected cells, characterized by a constricted actin ring beneath VP3^+^ or dsRNA^+^ cells (Fig. 1A). Thus, epithelial monolayers extrude RV-infected cells independently of the type of host cell receptor, because all strains elicited the same response. Because RV-A16, a major group, could be tracked from the earliest stages of infection using VP3 immunostaining, we used this strain for all future experiments.

Extrusion of RV-A16–infected cells was detectable at the earliest time point of 2 hours postinfection (hpi) (Fig. 1B), when the VP3 immunostaining appeared predominantly at the cell periphery, suggesting that viral attachment or early postattachment events may be sufficient to trigger extrusion. By contrast, at 24 hpi, VP3 localized throughout the cell during extrusion. While some noninfected cells extrude, as expected during homeostatic cell turnover, RV-A16–infected VP3^+^ cells were preferentially extruded, because infected monolayers exhibited increased cell extrusion rates, compared with mock-infected controls (Fig. 1C). We termed this epithelial response to RV VICE.

By 24 hpi, few VP3^+^ cells remained, steadily declining from ∼25% at 2 hpi to <5% at 24 hpi (Fig. 1D). Additionally, quantification of RV RNA by reverse transcription quantitative polymerase chain reaction (RT-qPCR) in both extruded and monolayer-associated cells (Fig. 1E) showed very few RV copies remaining in the infected monolayer by 24 hpi. Notably, both extruded and monolayer cells had equal RV RNA levels at 8 hpi, indicating that roughly half of the virus had been cleared by VICE by this time, consistent with immunostaining data in Fig. 1 (B and D). While RT-qPCR measures total viral RNA and immunofluorescence (IF) enables quantification of the percentage of infected cells, both methods showed the same trend in viral reduction following RV-A16 infection. The percentage of infected cells progressively declined as the rate of extrusion increased (Fig. 1F), supporting a direct role of VICE in RV clearance. Thus, 16HBE14o^−^ cells eliminate RV infection through VICE in the absence of other innate immune cells.

To confirm whether VICE occurs in real epithelial tissues, we infected ex vivo mouse lung slices with RVA1B, a minor RV strain that can infect mice. RVA1B induced high rates of extrusion in bronchiolar epithelia of mouse lung slices (at 24 hpi) (Fig. 1G). Because we lacked a structural RV antibody to RVA1B, we immunostained infected slices with dsRNA antibody at 24 hpi, at which point the extrusions were extensive enough to cause sheet detachment (Fig. 1G, yellow inset), similar to the damage resulting from broncho-constricted airways (*26*). The high extrusion rates may reflect a higher effective MOI, which is more difficult to calculate in tissue samples.

### An intact human airway epithelium clears RV-infected cells by VICE

**Fig. 2.**
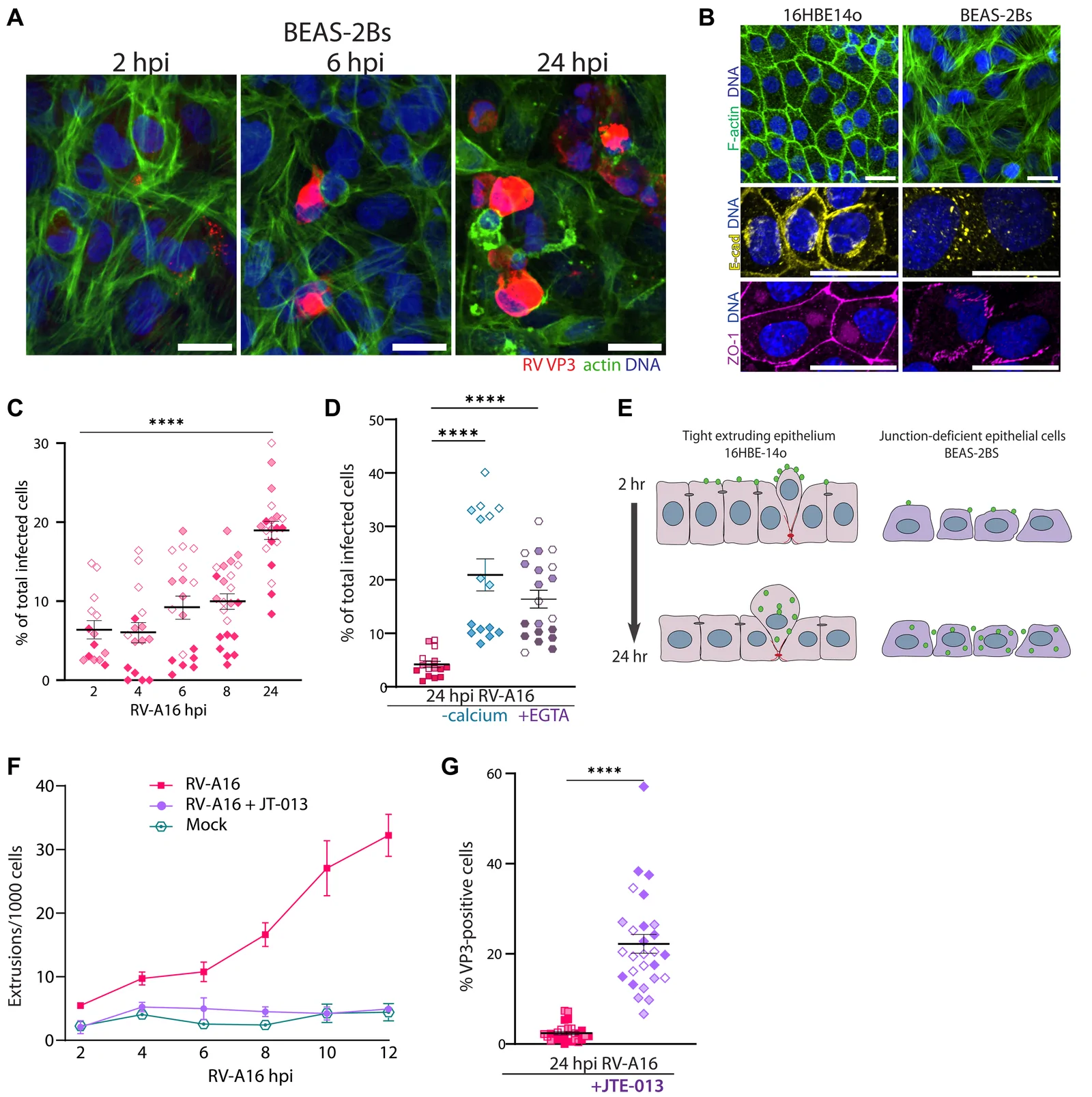
Intact airway epithelia clear RV-infected cells by VICE. (**A**) Confocal images of BEAS-2B cells infected with RV-A16 for 2, 6, and 24 hours. Scale bars, 25 μm. (**B**) Confocal projections of F-actin, E-cadherin, and ZO-1 staining in 16HBE14o^−^ and BEAS-2B monolayers. Scale bars, 10 μm. (**C**) Percentage of VP3^+^ infected BEAS-2B cells quantified every 2 hours after RV-A16 infection. (**D**) Percentage of VP3^+^ cells remaining at 24 hours in 16HBE14o^−^ monolayers with intact or disrupted junctions. (**E**) Schematic illustrating clearance of infected cells in extrusion-competent (16HBE14o^−^) versus nonextruding (BEAS-2B) epithelia. hr, hours. (**F**) Cell extrusion rates over time in RV-A16–infected 16HBE14o^−^ monolayers ± JTE-013. (**G**) Percentage of VP3^+^ cells remaining at 24 hours in RV-A16–infected 16HBE14o^−^ monolayers ± JTE-013. *n* ≥ 3 biological replicates, indicated by differentially shaded points, with mean and SEM. ****P < 0.0001 from one-way analysis of variance (ANOVA) with Tukey post hoc test (C) and Kruskal-Wallis test [(D) and (G)]. RV-A16 infections at an MOI of 3, unless otherwise indicated.

As we found that VICE could eliminate RV-infected cells from epithelial monolayers, we investigated why this phenomenon may not have been observed in previous studies. To do so, we infected BEAS-2B cells, a transformed human bronchial epithelial cell line widely used in RV studies (*27*). Unlike 16HBE14o^−^ monolayers (Fig. 1F), BEAS-2B cells could not extrude and failed to eliminate RV, resulting in RV accumulation over time (Fig. 2A). We reasoned that the inability of BEAS-2B cells to clear RV (Fig. 2A) might stem from their inability to form a tight epithelium with proper tight zonula occludins (ZO-1) and adherens junctions (E-cadherin) comparable with those observed in 16HBE14o^−^ monolayers (Fig. 2B). Thus, although BEAS-2B cells derive from bronchial epithelia, they behave more like mesenchymal cells (*28*).

To test whether the inability of BEAS-2B cells to eliminate RV infection by extrusion was due to their lack of proper cell-cell junctions, we disrupted 16HBE14o^−^ junctions by calcium removal or chelation with EGTA (fig. S1). Both approaches disrupted epithelial organization and prevented VICE-mediated RV clearance, resulting in RV accumulation comparable with that observed in BEAS-2B cells at 24 hpi (Fig. 2, C and D). These findings demonstrate that VICE-mediated RV clearance requires an extrusion-competent epithelial monolayer with intact cell-cell junctions (Fig. 2E).

To determine whether VICE-induced RV clearance requires canonical cell extrusion signaling, we next inhibited the sphingosine-1 phosphate receptor 2 (S1P2) signaling during RV infection. To extrude, cells secrete the lipid sphingosine-1-phosphate (S1P) that binds S1P2 to activate Rho to form and contract a basolateral actomyosin ring (*7*, *9*–*11*). Because most extrusion events observed following RV infection were VP3^+^ (Fig. 1C), we reasoned that we could score VICE rates over time using time-lapse phase imaging ± the S1P2 antagonist JTE-013. JTE-013 reduced VICE rates to levels comparable with mock-infected monolayers (Fig. 2F and movie S1 to S3), indicating that VICE requires canonical extrusion signaling. Inhibition of S1P2 signaling also impaired RV clearance from the monolayer at 24 hpi (Fig. 2G), demonstrating that epithelial extrusion contributes directly to removal of RV-infected cells. Together, these findings identify epithelial barrier organization and canonical S1P2 signaling as key regulators of VICE-mediated RV clearance.

### S1P2-dependent VICE promotes RV clearance in differentiated airway epithelia

**Fig. 3.**
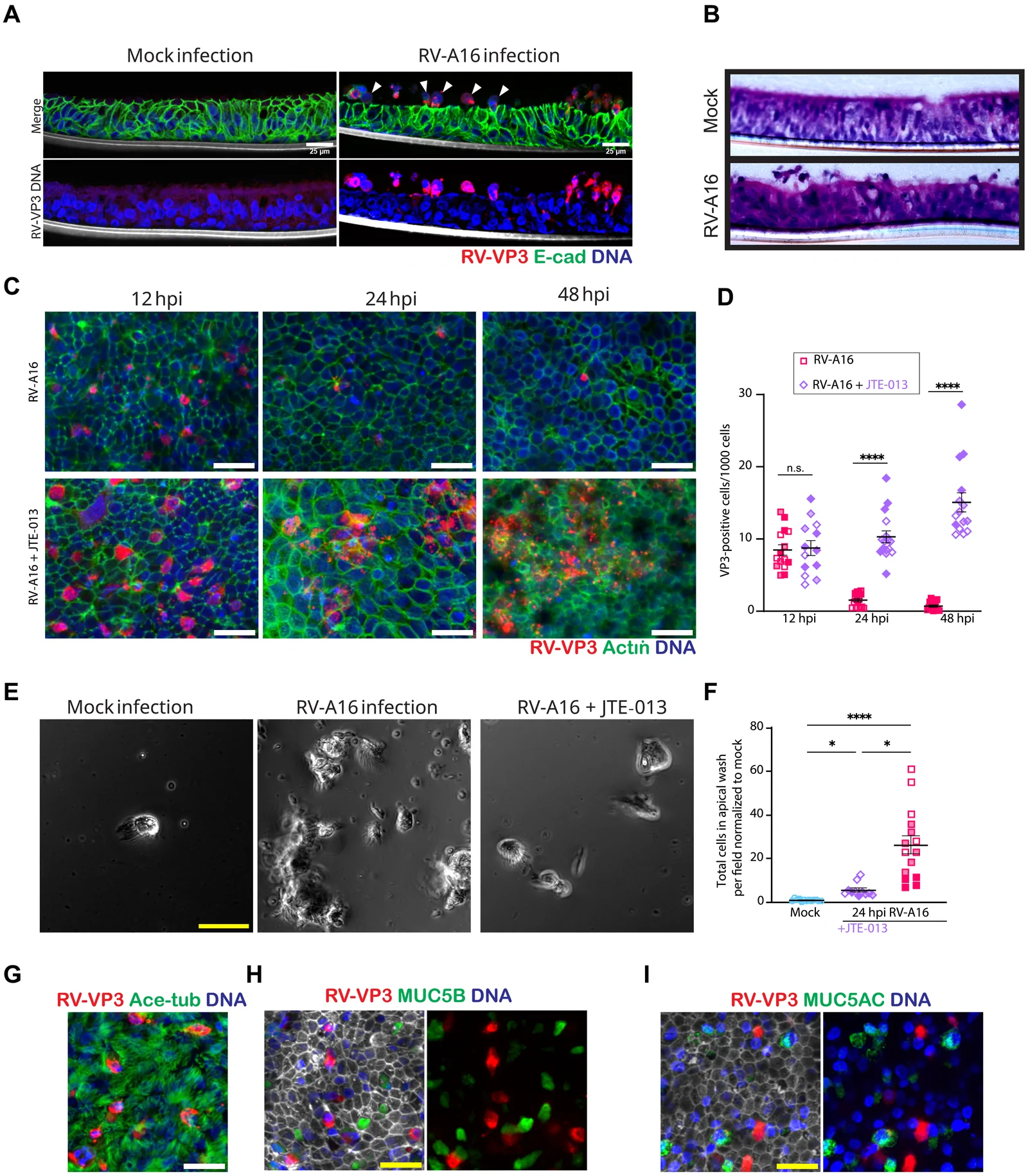
S1P2-dependent VICE promotes RV clearance in differentiated airway epithelia. (**A**) Confocal projections of fully differentiated primary ALI HAE mock infected or infected with RV-A16 for 24 hours and stained for RV-VP3, E-cadherin (E-cad), and DAPI. (**B**) Hematoxylin and eosin (H&E) staining of mock-infected and RV-A16–infected ALI cultures at 24 hpi. (**C**) Confocal projections of ALI cultures infected with RV-A16 ± JTE-013 at 12, 24, and 48 hpi and stained for RV-VP3, F-actin, and DAPI. (**D**) Quantification of RV-VP3^+^ cells per 1000 cells over time in RV-A16–infected ALI cultures ± S1P2 antagonist JTE-013. (**E**) Phase-contrast images of apical washes from mock-infected, RV-A16–infected, or RV-A16 + JTE-013–treated ALI cultures at 24 hpi. (**F**) Quantification of cells from apical washes of mock-infected, RV-A16–infected, and RV-A16 + JTE-013–treated ALI cultures at 24 hpi. (**G**) Confocal projections of RV-A16–infected ALI cultures stained for RV-VP3, acetylated tubulin (Ace-tub), and DAPI, demonstrating infection of ciliated epithelial cells. (**H** and **I**) Confocal projections of RV-A16–infected ALI cultures stained for RV-VP3 with MUC5B (H) or MUC5AC (I) and DAPI. *n* ≥ 3 biological replicates, indicated by differently shaded points, shown as means ± SEM. Statistical analysis performed using Mann-Whitney test (D) and Kruskal-Wallis test with Dunn’s multiple comparisons test (F). *P < 0.05 and ****P < 0.0001. White arrowheads indicate extruding infected cells. Scale bars, 25 μm. n.s., not significant.

To determine whether VICE occurs in fully differentiated airway epithelia, we infected primary HAE cultures (Table 1) grown at an air-liquid interface (ALI) with RV-A16. Like 16HBE14o^−^ monolayers, RV-A16 infection induced extrusion of RV-VP3^+^ cells from the epithelial layer (Fig. 3, A and B). Most infected cells were eliminated by 24 to 48 hpi (Fig. 3, C and D), with the few remaining cells being actively extruded (white arrows in Fig. 3C). Live imaging of apical washes revealed beating ciliated cells following RV-A16 infection (movie S4), demonstrating that extruded epithelial cells remain viable following extrusion. JTE-013 confirmed that VICE in ALI cultures also requires S1P2 signaling (Fig. 3, C and D) and markedly reduced the number of extruded cells recovered from an apical wash at 24 hpi (Fig. 3, E and F). Untreated RV-infected cultures progressively reduced RV-VP3^+^ cells by 24 to 48 hpi, whereas those with JTE-013 accumulated significantly higher numbers of infected cells over time (Fig. 3, C and D).

**Table 1.** Description of bronchial airway epithelial cell donors. All donors were healthy nonsmokers. Cultures were differentiated at air-liquid interface (ALI) conditions for at least 30 days before infection experiments. Donor 1 ALI cultures were provided by A. A. Nikonova (Imperial College London).

Donor	Sex	Age (years)	Source
1	Female	55	Gifted culture
2	Male	35	Epithelix
3	Male	17	Epithelix
4	Male	15	Epithelix

To identify which epithelial cell types induce VICE, ALI cultures were costained with markers of differentiated airway epithelial cells. RV-VP3^+^ cells predominantly colocalized with acetylated tubulin, confirming infection and extrusion of ciliated epithelial cells (Fig. 3G) with only occasional RV-VP3^+^ costaining with MUC5B^+^ and MUC5AC^+^ goblet cells (Fig. 3, H and I). This may reflect that most cells in ALI cultures are ciliated or that RV preferentially infects ciliated epithelial cells (*22*–*24*). Together, these findings demonstrate that differentiated airway epithelia clear RV infection through S1P2-dependent VICE.

### RV induces two waves of cell extrusion: A live and an apoptotic one

**Fig. 4.**
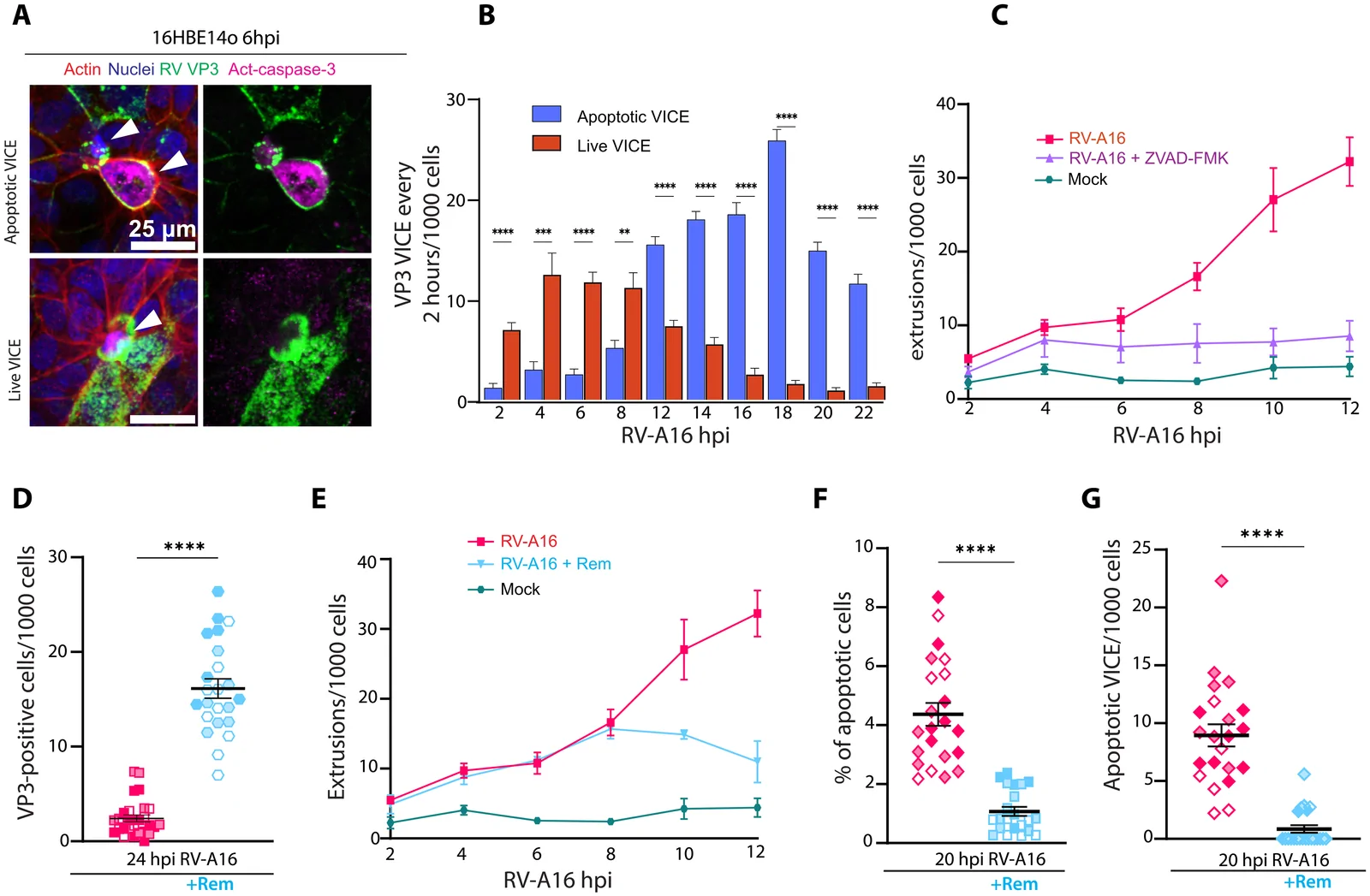
RV induces two waves of cell extrusion: a live and an apoptotic one. (**A**) Confocal images of apoptotic and nonapoptotic extrusions at 6 hpi, stained for actin (red), nuclei (blue), VP3 (green), and active caspase-3 (Act-caspase-3; magenta). White arrowheads indicate extruding cells, quantified over time in (**B**). (**C**) Extrusion rates in control or z-VAD-FMK–treated RV-A16–infected cells. (**D**) Percentage of VP3^+^ cells at 24 hours ± remdesivir (Rem). (**E**) Extrusion rates ± remdesivir, every 2 hours for 12 hours by live-cell imaging. (**F**) Apoptosis ± remdesivir. (**G**) Apoptotic extrusions ± remdesivir. *n* ≥ 3 biological replicates, indicated by differentially shaded points, with mean and SEM using an MOI of 3. **P < 0.01, ***P < 0.001, and ****P < 0.0001 by two-tailed *t* test (B), Kruskal-Wallis test (D), Welch’s *t* test (F), and Mann-Whitney test (G).

While all apical extrusion pathways require the canonical S1P signaling tested above, different stimuli can trigger this signaling via different upstream pathways (*29*). To maintain homeostatic cell densities, most cells extrude while alive in response to crowding via Piezo1 (*10*); however, apoptotic stimuli can also induce extrusion to ensure that dying cells do not compromise the integrity of the monolayer (*7*, *30*). To investigate whether RV induces apoptotic or live-cell extrusion, we infected 16HBE14o^−^ monolayers with RV-A16 and immunostained for the viral structural protein VP3, active caspase-3 (a marker of apoptosis), and phalloidin (actin) to visualize extrusion rings. We found that VICE was predominantly nonapoptotic during the first 8 hpi, shifting to mainly apoptotic extrusions for the next 12 hours (Fig. 4, A and B). These two separate waves of live and apoptotic extrusion likely account for the two peaks of total VICE over 24 hours, seen in Fig. 1C.

Given that apoptotic extrusion requires caspase activity (*30*), we tested whether the second wave of VICE requires caspases by treating infected 16HBE14o^−^ cells with z-VAD-FMK, an irreversible pan-caspase inhibitor (*31*, *32*). Time-lapse imaging revealed markedly reduced extrusion rates from 8 hours onward in z-VAD-FMK–treated cells, compared with those in untreated controls, without affecting the first wave of extrusion (Fig. 4C). Because RV replication typically occurs at 8 hpi (*2*), we tested whether the second apoptotic VICE wave requires RV replication using remdesivir, a cell-permeable monophosphate that blocks viral RNA replication in RV, Ebola virus, Middle East Respiratory Syndrome Coronavirus 2, severe acute respiratory syndrome coronavirus, and severe acute respiratory syndrome coronavirus 2 (*33*, *34*). We confirmed that remdesivir blocks dsRNA synthesis following RV infection in HeLa-H1 cells (fig. S2). Notably, remdesivir treatment throughout the course of infection greatly reduced RV clearance through VICE at 24 hpi (Fig. 4D). This unexpected reduction in RV clearance may account for why remdesivir is not as efficacious as expected. Live imaging showed that VICE rates in RV-A16–infected 16HBE14o^−^ cells treated with remdesivir were like untreated cells during the first 8 hours but declined significantly thereafter (Fig. 4E). Additionally, remdesivir reduced apoptosis in RV-infected 16HBE14o^−^ monolayers, compared with untreated infected cells (Fig. 4F), leading to fewer apoptotic extrusions (Fig. 4G). Thus, viral replication triggers the second apoptotic wave of VICE.

### The first VICE wave is mechanical and independent of dynamin-dependent RV endocytosis

**Fig. 5.**
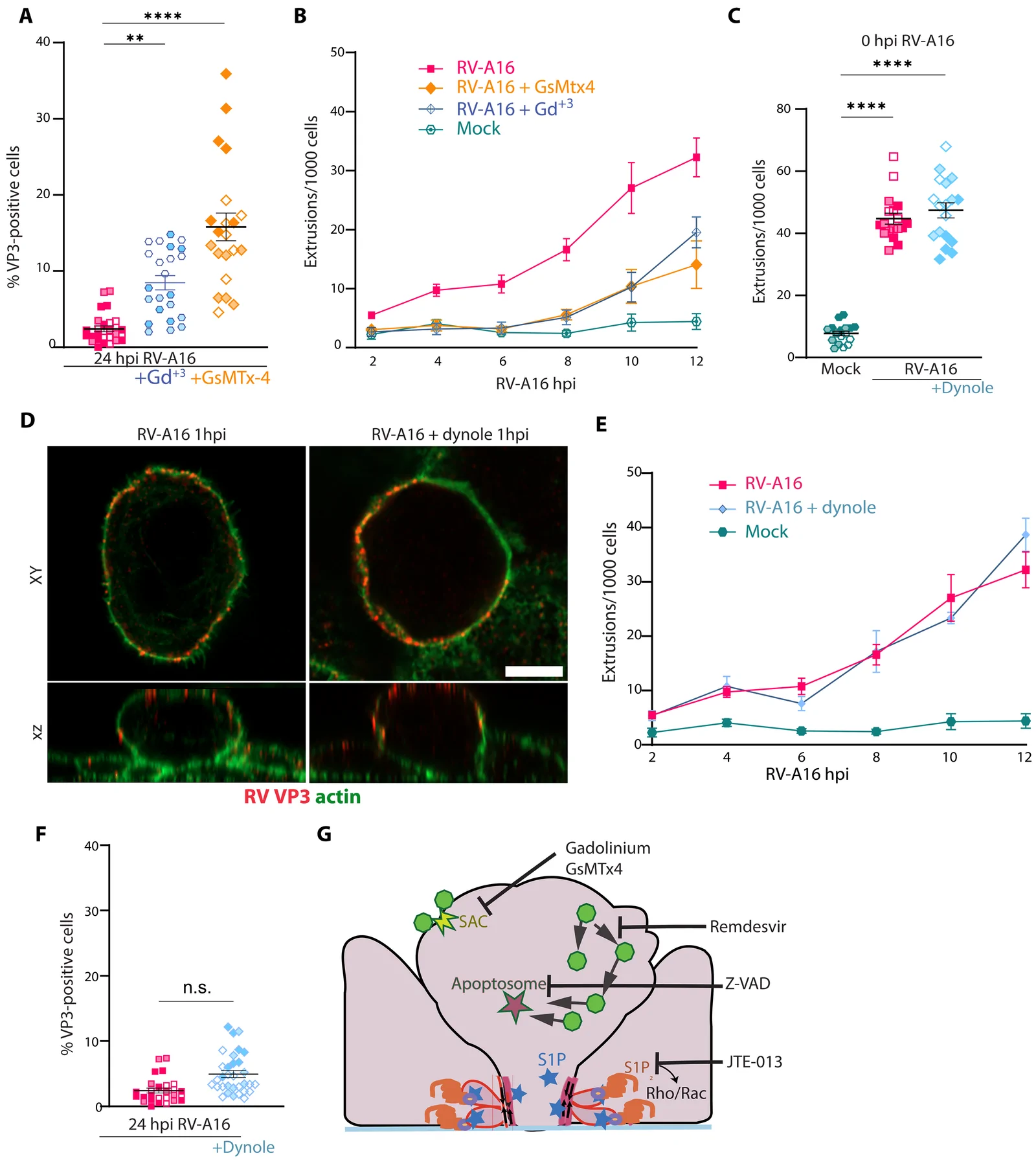
The first extrusion wave is mechanical and independent of dynamin-dependent RV endocytosis or cell death. (**A**) Percentage of VP3^+^ cells remaining at 24 hpi ± Gd^3+^ or GsMtx4. (**B**) Extrusion rates measured every 2 hours ± Gd^3+^/GsMtx4-treated monolayers. (**C**) Total extrusion events at 0 hpi ± dynole 34-2. (**D**) Confocal XY and XZ slices at 1 hpi ± dynole 34-2. Scale bar, 10 μm. (**E**) Extrusion rates ± dynole 34-2 every 2 hours. (**F**) Percentage of VP3^+^ cells remaining at 24 hours ± dynole 34-2. (**G**) Schematic showing inhibitors used in the study: Gd^3+^ and GsMtx4 block stretch-activated/TRP channels, z-VAD-FMK blocks apoptosis, remdesivir blocks viral replication, and JTE-013 blocks S1P_2_ receptor. *n* ≥ 3 biological replicates, indicated by differentially shaded points, with mean and SEM using an MOI of 3. Not significant (n.s.), *P* > 0.05; ** *P* < 0.01; and ****P < 0.0001 by Kruskal-Wallis test.

Because the first VICE wave is predominantly live-cell extrusion, we tested whether SACs, which typically control live-cell extrusion during homeostasis upstream of S1P2 signaling, control this first wave. To do so, we infected 16HBE14o^−^ in the presence or absence of gadolinium (Gd^+3^), a generic SAC blocker or GsMTx4, a tarantula venom peptide toxin that inhibits the transient receptor potential channels TRPC1, TRPC6, and Piezo1 (*35*, *36*). Both Gd^+3^ and GsMTx4 significantly reduced RV clearance by 24 hpi (Fig. 5A), albeit to a lesser extent than blocking S1P2 with JTE-013. Live imaging demonstrated that both Gd^+3^ and GsMtx4 reduced VICE during the first 8 hours of infection (Fig. 5B), indicating that SACs control the first wave of VICE, accounting for reduced RV clearance. In both cases, GsMTx4 and Gd^+3^ reduced VICE and virus clearance (Fig. 5, A and B), confirming the tight correlation between VICE and RV clearance. These results confirm that VICE occurs in at least two waves, with the first live-cell extrusion wave being controlled by SACs and the second apoptotic cell extrusion wave dependent on viral replication.

Given that RV localized to cell borders during VICE at the earliest time point measured (2 hpi, Fig. 1B), we investigated whether VICE might be activated before virus internalization. In our standard protocol, RV is adsorbed for 1 hour at room temperature, after which the unbound virus is washed off, and cells are incubated at 37°C (fig. S3A). We therefore tested whether events occurring during adsorption period are sufficient to elicit VICE. At 0 hpi, adsorption induced markedly higher rates of VICE than those observed at 2 hpi (∼45 versus ∼5 extrusions per 1000 cells; Fig. 5, A and E). Confocal imaging at this time point confirmed that RV remained at the plasma membrane during VICE (Fig. 5D), with the single *Z*-stacks of +dynole condition shown in fig. S3D.

To more specifically test whether VICE requires dynamin-mediated endocytosis (*37*), we infected 16HBE14o^−^ monolayers with RV-A16 ± dynole 34-2, a cell-permeable allosteric inhibitor of dynamin-mediated endocytosis (*38*). A transferrin uptake assay confirmed that dynole 34-2 inhibits dynamin-dependent endocytosis in our system (fig. S3B). Dynole 34-2 treatment produced the same high rates of VICE and the same peripheral VP3 staining as observed during the adsorption step (Fig. 5, C and D). Additionally, inhibiting endocytosis with dynole 34-2 did not alter VICE rates over time (Fig. 5E) or significantly reduce RV-VP3^+^ at 24 hpi, compared with control infections (Fig. 5F). While RV localizes predominantly to plasma membrane during VICE between 2 and 8 hpi, the few extruding infected cells observed at 24 hpi in dynole 34-2–treated monolayers had internalized virus (by VP3 staining) (fig. S3C), potentially due to internalization by macropinocytosis (*39*). Thus, epithelia use two strategies to ensure extrusion can eliminate RV infections: by activating live-cell extrusion before the virus completely enters the cell, which is potentially mechanically activated, and by apoptotic cell extrusion in cells where RV has replicated (Fig. 5G).

### Extruded virus-infected cells can infect other cells

**Fig. 6.**
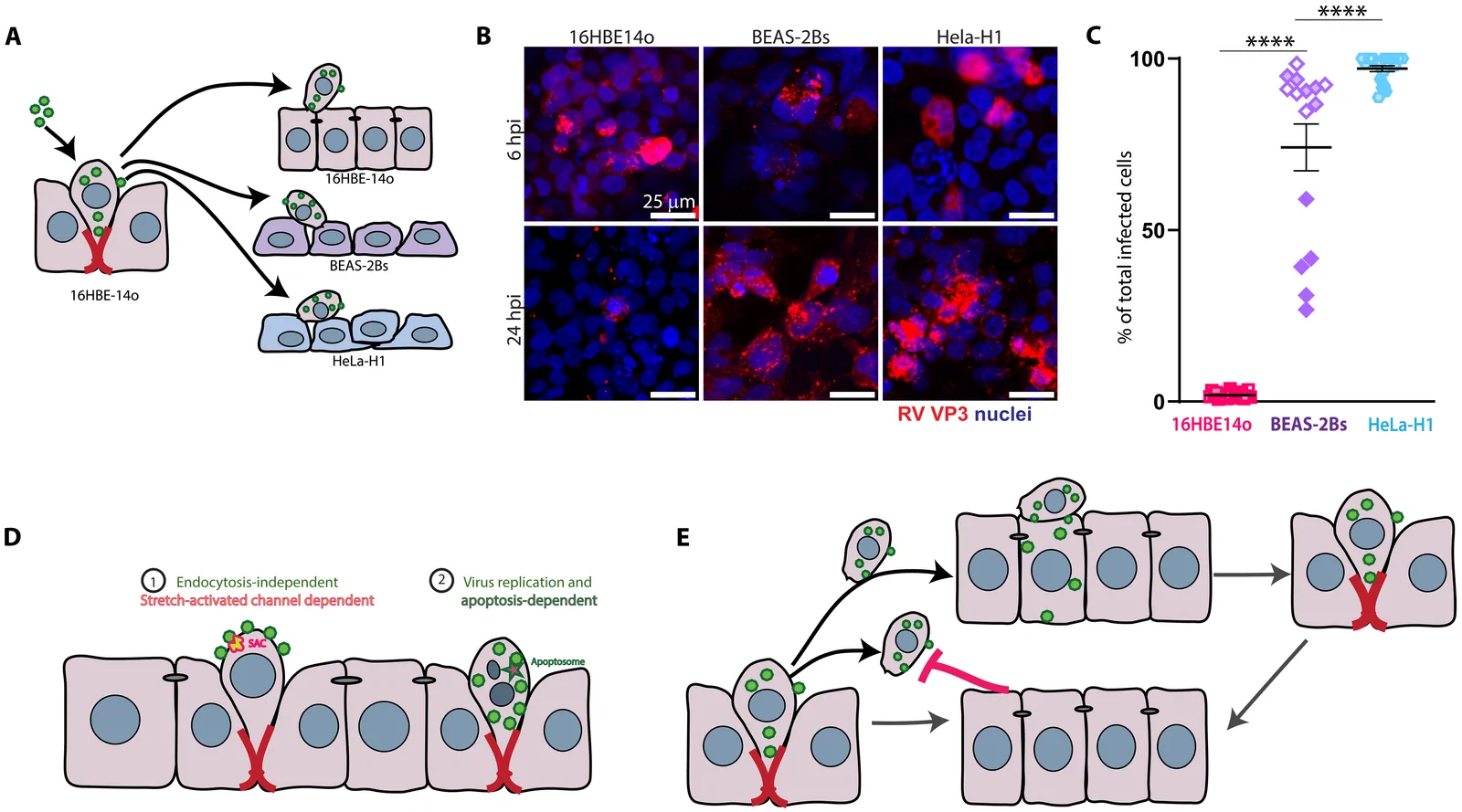
Extruded RV-infected cells can infect new monolayers. (**A**) Schematic showing experimental design: Extruded infected cells from RV-A16–infected monolayers are added to naïve 16HBE14o^−^, BEAS-2B, or HeLa-H1 monolayers. (**B**) Confocal projections of each monolayer at 6 and 24 hours after infected, extruded cell addition, quantified in (**C**). (**D** and **E**) Models illustrating that RV-A16–infected monolayers clear infection via extrusion (D) and that extruded cells can initiate infection in fresh naïve monolayers (E). *n* ≥ 3 biological replicates, indicated by differentially shaded points, with mean and SEM using an MOI of 3. ****P < 0.0001 by Kruskal-Wallis test.

While epithelia eliminate RV by VICE, the extruded cells remain laden with virus and may retain infectious potential. To test whether the cells extruded from VICE could infect naive cells, we collected extruded cells at 16 hpi, washed them with medium, and added them to fresh BEAS-2B, HeLa-H1, or 16HBE14o^−^ monolayers, incubating for 24 hours (Fig. 6A). We found that extruded VICE cells resulted in naïve monolayers becoming infected within 6 hours of addition and that infection progressively increased in BEAS-2B and HeLa-H1 cells, such that most cells were infected by 24 hpi. By contrast, 16HBE14o^−^ monolayers reduced RV burden by VICE within 24 hours, as expected (Fig. 6, B and C).

Notably, ∼100% of BEAS-2B cells became infected following exposure to the virus-laden extruded cells (Fig. 6C), compared with only ∼20% with purified RV by 24 hpi (Fig. 2H). While the extruded infected cells likely contain higher viral loads than cell-free virus preparations, the relative MOI between these conditions cannot be directly compared. Thus, although we cannot determine whether extruded infected cells are intrinsically more infectious than purified virus, our findings demonstrate that virus-laden extruded cells remain infectious and can infect naïve epithelial monolayers.

## DISCUSSION

Here, we found that epithelia rapidly clear RV by inducing VICE. VICE endows airway epithelia with a rapid protective response without the need for other innate or adaptive immune cells (Figs. 1 and 3). Previous studies have described shedding or sloughing of infected airway epithelial cells following RV, RSV, and measles virus infection of respiratory epithelia (*21*–*24*, *40*, *41*) and enterovirus infection of gut epithelia (*20*). More recently, measles virus–infected epithelia induce epithelial-to-mesenchymal transition, which may be due to live basal cell extrusion (*42*). However, these studies did not establish the molecular signaling mechanisms driving RV-induced epithelial extrusion. Here, we show that VICE occurs through two mechanistically distinct waves: an early SAC-dependent phase that occurs independently of dynamin-dependent viral entry or replication (Fig. 5), followed by a later viral replication-dependent apoptotic phase (Fig. 4). Both waves depend on S1P signaling through the S1P2 receptor in human epithelial cell lines and a primary differentiated ALI (Figs. 1 and 3). Although highly efficient, the response is a double-edged sword, eliminating infected cells but facilitating rapid dissemination of infectious virus (Fig. 6E).

The elimination of virus by the epithelium alone is typically overlooked in virology, likely because most studies use cell lines incapable of forming mature epithelial junctions, which are required for extrusion (Fig. 2). This has led the field to focus predominantly on blood-based innate and adaptive immune responses while underappreciating the epithelium’s intrinsic role in innate immunity. The growing use of primary ALI cultures, however, has brought greater attention to cell shedding as a direct epithelial response to viral infection. We propose that the VICE pathway described here accounts for how differentiated airway epithelia resist RV infection and reduce viral burden independently of immune cells (*43*, *44*). Elucidating the signaling and mechanisms underlying VICE is, therefore, critical to understanding this fundamental first-line innate immune defense against respiratory viruses and other pathogens.

It is important to note that VICE may not target all viruses. An electron microscopy study reporting no shedding response to parainfluenza virus suggests the same for that pathogen (*24*). Identifying the signaling and mechanisms driving VICE is, therefore, important for understanding how some viruses may evade or exploit this pathway during infection (*25*) and could inform previously unidentified therapeutic strategies aimed at modulating epithelial VICE responses to promote viral clearance.

Last, our finding that extruded cells from VICE can efficiently infect other cells suggests two important points (Fig. 6E). One, extruded virus-laden cells can infect naïve monolayers efficiently, suggesting a physiologically relevant spread route for viral dissemination, consistent with the findings by Moshiri *et al.* (*20*). Two, while these virus-laden cells can infect new cells, the infected 16HBE14o^−^ monolayers do not appear to become reinfected from the virus-laden extruded cells floating in their medium, suggesting a previously unknown form of epithelia-specific learned innate immune response, which will need further investigation. This finding makes practical sense as unchecked continuous reinfection and VICE within the same epithelium would rapidly destroy it. Identifying such a mechanism could lead to new therapeutic avenues aimed at preventing viral infections altogether, rather than merely managing their symptoms.

Our findings that epithelia can eliminate RV infection without the assistance of blood-based immunity have many implications. Because extrusion is a primordial process, conserved in *Trichoplax*, sea sponges, and sea anemones (*45*–*47*), VICE may account for how these epithelial-based organisms have such robust immune systems. Understanding this conserved, intrinsic form of innate immunity will be of great importance to our management of virus spread and symptoms.

## MATERIALS AND METHODS

### Cell culture

16HBE14o (Merck, SCC150), HeLa-H1 [American Type Culture Collection (ATCC), CRL-1958], and BEAS-2B cells were grown in their respective growth medium (Table 2) supplemented with 10% fetal bovine serum (FBS; 10270106) and 1% penicillin/streptomycin (P/S; 15070063) in a cell culture incubator at 37°C with 5% CO_2_. For all experiments, cells were seeded in growth medium and cultured until confluent. At confluency, the growth medium was removed, and cells were washed twice with infection medium (Table 2) and incubated overnight in the same medium. All cell lines tested negative for mycoplasma contamination in periodic tests (Sartorius, 20-700-20).

**Table 2.** List of reagents and buffers. BSA, bovine serum albumin; CCK-8, cell counting kit-8; DAPI, 4′,6-diamidino-2-phenylindole; DMEM, Dulbecco’s modified Eagle’s medium; DMSO, dimethyl sulfoxide; DPBS, Dulbecco’s phosphate-buffered saline; dNTP, deoxynucleotide triphosphate; FBS, fetal bovine serum; GAPDH, glyceraldehyde-3-phosphate dehydrogenase; HBSS, Hanks’ balanced salt solution; MEM, minimum essential medium; P/S, penicillin/streptomycin; RNAse, ribonuclease.

Cell culture growth medium					
Cell/tissue	Growth medium		Catalog number		Supplier
HeLa-H1	DMEM		D6429		Sigma-Aldrich
BEAS-2B	DMEM		31966-024		Gibco
16HBE14o^−^	MEM		31095-029		Gibco
List of medium/buffer recipes					
Buffer/medium	**Recipe**				**Cell line**
HBE growth medium	MEM supplemented with 10% FBS and 1% P/S				16HBE14o^−^
BEAS-2B growth medium	DMEM (Gibco) supplemented with 10% FBS and 1% P/S				BEAS-2B
HeLa growth medium	DMEM (Sigma-Aldrich) supplemented with 10% FBS, 2% 1 M Hepes, 1% GlutaMAX, and 1% NaHCO_3_				Hela-H1
Infection medium	Growth medium supplemented with 2% FBS, 2% 1 M Hepes, 1% GlutaMAX, and 1% NaHCO_3_				Various
IF blocking buffer	2% BSA, 0.1% Triton X-100, and 0.1% sodium azide				Various
Freezing medium	10% DMSO and 90% FBS				Various
List of antibodies					
Protein/epitope	**Antibody type**	**Host**	**Catalog number**	**Supplier**	**Dilution**
VP3	Primary	Mouse	MA5-18250	Invitrogen	1:100
dsRNA	Primary	Mouse	MABE1134	Sigma-Aldrich	1:100
MDA5	Primary	Rabbit	NBP1-76760	Novus	1:100
Active caspase-3	Primary	Rabbit	9669S	Cell Signaling Technology	1:100
Piezo1	Primary	Rabbit	NBP1-78537	Novus	1:200
E-cadherin	Primary	Rabbit	3195S	Cell Signaling Technology	1:200
ZO-1	Primary	Rabbit	617300	Invitrogen	1:200
Acetylated-α tubulin	Primary	Rabbit	D2093	Cell Signaling Technology	1:100
MUC5AC	Primary	Rabbit	E3O9I	Cell Signaling Technology	1:100
MUC5B	Primary	Rabbit	E4Y5Z	Cell Signaling Technology	1:100
Influenza nucleocapsid	Primary	Rabbit	Gtx125989	GeneTex	1:100
Phalloidin 488	Secondary	N/A	A12379	Invitrogen	1:200
Phalloidin 568	Secondary	N/A	A12380	Invitrogen	1:200
Phalloidin 647	Secondary	N/A	A22287	Invitrogen	1:200
488 Anti-mouse	Secondary	Goat	A32723	Invitrogen	1:200
568 Anti-rabbit	Secondary	Goat	A11011	Invitrogen	1:200
568 Anti-mouse	Secondary	Goat	A11004	Invitrogen	1:200
647 Anti-rabbit	Secondary	Goat	A32733	Invitrogen	1:200
647 Anti-rabbit	Secondary	Goat	A21235	Invitrogen	1:200
DAPI	N/A	N/A	D1306	Invitrogen	1:1000
List of reagents					
Reagent		**Catalog number**		**Supplier**	
MEM		31095-029		Gibco	
DMEM (Sigma-Aldrich)		D6429		Sigma-Aldrich	
DMEM (Gibco)		31966-024		Gibco	
FBS		10270106		Gibco	
P/S		11580626		Gibco	
DPBS		14190250		Gibco	
Trypsin		25300-096		Gibco	
HBSS		14025		Gibco	
PFA		047317.9M		Thermo Fisher Scientific	
EGTA		324626		Merck	
Triton X-100		X100RS		Sigma-Aldrich	
BSA		A9418		Sigma-Aldrich	
Sodium azide		S8032		Sigma-Aldrich	
ProLong Gold		P36930		Thermo Fisher Scientific	
dNTP		DNTP100-1KT		Sigma-Aldrich	
Random hexamer		SO142		Thermo Fisher Scientific	
RiboLock RNAse Inhibitor		EO0382		Thermo Fisher Scientific	
RevertAid H Minus RT		EP0452		Thermo Fisher Scientific	
TRIzol		15596026		Thermo Fisher Scientific	
GlycoBlue		AM9516		Thermo Fisher Scientific	
Luna buffer		M3004E		New England Biolabs	
Chloroform		C/920/15		Fisher Scientific	
Dynole		AB120463		Abcam	
DMSO		276855		Merck	
Low melt agarose		Bp1360-100		Sigma-Aldrich	
Rat collagen		C3867		Sigma-Aldrich	
Trypan blue		T8154-100ML		Merck	
Nail varnish		N/A		Boots	
CCK-8		96992		Merck	
MTT assay growth kit		CT02		Merck	
List of drugs or inhibitors used and in RV-A16 infection					
Drug/inhibitor	**Concentration**		**Target**	**Catalog number**	**Supplier**
JTE-013	10 μM		S1P2 receptor	J4080	Sigma-Aldrich
GsMtx4	50 μM		Piezo1, TRPC1, and TRPC6	08GSM001	Smartox
z-VAD-FMK	50 μM		Pan-caspase inhibitor (apoptosis)	V116-2MG	Merck
Remdesivir	25 μM		Viral replication	AB120463-1001	StraTech
GdCl3	10 μM		Stretch-activated channels	G7532	Sigma-Aldrich
Dynole	10 μM		Dynamin-dependent endocytosis	AB120463-1001	Abcam
Reaction components used for cDNA synthesis					
Reagent				**Volume/reaction**	
Random hexamer primers (10 mM)				0.5 μl	
5× Buffer RT				2 μl	
dNTP mix (10 mM each dNTP)				1 μl	
RiboLock RNAse inhibitor (40 U/μl)				0.25 μl	
RevertAid H Minus RT (200 U/μl)				0.5 μl	
RNA template (300 ng)				Varies	
Total reaction volume				10 μl	
Primers and probes used for qPCR					
Gene				**Primer/probe/set**	
Rhinovirus forward (900 nM)				GTGAAGAGCCSCRTGTGCT	
Rhinovirus reverse (900 nM)				GCTSCAGGGTTAAGGTTAGCC	
Rhinovirus probe				TGAGTCCTCCGGCCCCTGAATG	
GAPDH				Hs02786624_g1	
SDHA				HK-DD-hu-900	

### RV stock production and titration

Viral stocks of RVA1B, RVA2, RVB14, and RV-A16 (all from ATCC) were generated in HeLa-H1 cells and their titers were determined by measuring 50% tissue culture infectious dose (TCID_50_), as described previously (*48*). Briefly, cells at ∼90% confluency were washed twice with Dulbecco’s modified Eagle’s medium infection medium and inoculated with the virus diluted in infection medium. After 1 hour of gentle agitation at room temperature, cultures were incubated at 37°C and 5% CO_2_ until ∼90% cytopathic effect (CPE) was observed. Cells were then dislodged, subjected to two freeze-thaw cycles at −80°C, and clarified by centrifugation (4000 rpm, 15 min, 4°C). The supernatant was filtered (0.2 μm; catalog no. S2GPU01RE) and stored in aliquots at −80°C.

For TCID_50_ determination, HeLa-H1 cells were seeded at 0.5 × 10^5^ cells/ml (150 μl per well, 96-well plate). Undiluted virus or 10-fold serial dilutions were added, and plates were incubated for 4 to 5 days at 37°C and 5% CO_2_. CPE was scored microscopically, and TCID_50_/ml values were calculated using the Spearman-Kärber method (*49*).

### RV infection of cell cultures

Cells were seeded at equal densities into four- or eight-well ibidi chambers or 24-well plates with size-1.5 coverslips and cultured at 37°C with 5% CO_2_ until ∼95% confluency, followed by serum-starvation overnight in medium containing 2% FBS.

Drug cytotoxicity was evaluated using MTT (Merck, CT02) or cell counting kit-8 (Merck, 96992) assays, according to the manufacturer’s instructions. For experiments with remdesivir or dynole 34-2, cells were preincubated with the drug for 2 hours at 37°C with 5% CO_2_ before infection. Viral stocks were diluted in infection medium to achieve a MOI of 3, with inoculum volumes adjusted for each format (150 μl for 24-well plates, 150 μl for 8-well chambers, 500 μl for 6-well plates, and 200 μl for 4-well chambers). For drug treatments, the inoculum contained the drug at the corresponding concentration. Cells were inoculated for 1 hour at room temperature with gentle agitation, washed three times with growth medium, and maintained at 37°C and 5% CO_2_ in fresh medium with or without drug.

Cultures were harvested at 0 to 96 hpi for downstream applications, including enzyme-linked immunosorbent assay from supernatants, IF from coverslips, or protein/RNA extraction. ibidi chambers or glass-bottom 24-well plates were used for time-lapse imaging.

### Infection of primary HAE cultures

Differentiated primary human bronchial airway epithelial cells grown at an ALI were maintained with basolateral MucilAir culture medium (EP04MM; Epithelix) only and weekly apical mucus washes. Before infection, the apical surface was washed with warm Dulbecco’s phosphate-buffered saline (DPBS). HAE cultures were infected apically with RV-A16 (10^4^ TCID_50_ in 100 μl) for the indicated times. Following adsorption, the inoculum was removed and the apical surface washed once with warm DPBS. For inhibition experiments, cultures were basolaterally pretreated with JTE-013 for 1 hour before infection, and the inoculum additionally contained the inhibitor.

Apical washes containing extruded cells were collected at 24 hpi for live imaging or downstream analysis. Cultures were fixed in 4% paraformaldehyde (PFA), and membranes were excised from inserts and processed as whole mounts or paraffin-embedded sections (4 μm) for hematoxylin and eosin (H&E) staining and IF.

### Disruption of cell junctions

#### Calcium-free medium

Cells were seeded at equal densities and cultured in HBE growth medium without calcium (minimum essential medium, no calcium; Thermo Fisher Scientific, 11380037) supplemented with 2% GlutaMAX, 10% FBS, and 1% P/S for 36 hours. The medium was then replaced with calcium-free HBE infection medium and incubated overnight. Infection was performed at MOI of 3 in calcium-free medium for 1 hour at room temperature with gentle agitation. Cells were washed three times and cultured in calcium-free growth medium at 37°C and 5% CO_2_ until harvest.

#### EGTA treatment

Cells were grown to ∼90% confluency and serum-starved overnight in infection medium. The following day, cells were preincubated in infection medium containing 1 mM EGTA. Infection was performed at MOI of 3 in EGTA-supplemented infection medium for 1 hour at room temperature with gentle agitation. After inoculum removal, cells were washed three times and maintained in growth medium containing 1 mM EGTA at 37°C with 5% CO_2_ until harvest.

Cells were grown to ∼90% confluency and serum-starved overnight in infection medium. The next day, cells were preincubated in infection medium containing 1 mM EGTA and then inoculated at an MOI of 3 in EGTA-supplemented medium for 1 hour at room temperature with gentle agitation. Following inoculum removal, cells were washed three times and cultured in growth medium containing 1 mM EGTA at 37°C and 5% CO_2_ until harvest.

### IF staining

Cells grown on coverslips were fixed with 4% PFA at designated time points and rinsed three times with DPBS. After blocking for 1 hour at room temperature in IF blocking buffer (Table 2), cells were incubated overnight at 4°C with primary antibodies (Table 2). Following three rinses and three 10-min washes in DPBS, cells were incubated with secondary antibodies (Table 2) for 2 hours at room temperature. Nuclei were counterstained with DAPI (1:1000, v/v) for 10 min, washed, mounted in ProLong Gold (Table 2), and cured for 24 hours before imaging.

For paraffin-embedded sections, slides were baked at 60°C for 1 hour, deparaffinized in xylene, rehydrated through graded ethanol washes, and subjected to hot water antigen retrieval before immunostaining. For precision-cut lung slices (PCLSs), the procedure was identical, except secondary antibodies were incubated overnight at 4°C, and washes were extended to three 30-min steps.

### Harvesting extruded cells

16HBE14o cells infected with RV-A16 were processed for recovery of extruded cells. Culture medium was collected into a 15-ml Falcon tube, and the remaining monolayer was treated with 500 μl of trypsin at room temperature for ∼2 min, with detachment monitored by light microscopy. Trypsinization was quenched with 2 ml of HBE infection medium, and detached cells were pooled with the collected medium. Suspensions were centrifuged at 1200 rpm for 5 min at 4°C to pellet extruded cells, which were washed three times and resuspended in infection medium.

### Microscopy

Live-cell phase contrast and IF imaging were performed on a Nikon Eclipse Ti2 microscope. For time-lapse phase imaging, a Plan Fluor 20× Ph1 DLL numerical aperture (NA) 0.50 objective, a Photometrics Iris 15 16-bit camera, and a CoolLED pE-4000 light source were used, controlled by NIS-Elements software (Nikon, v5.30.02). For IF imaging, Plan Fluor 20×, 40× water-immersion, or 60× NA 1.40 oil-immersion objectives were used in combination with an iXon 888 Andor 16-bit camera, a Yokogawa CSU-W1 spinning disk confocal unit, and Toptica Photonics laser excitation, controlled by NIS-Elements (Nikon, v5.21.03).

### RNA extraction and cDNA synthesis

RNA from extruded or monolayer cells was isolated using TRIzol Reagent (Thermo Fisher Scientific) with minor modifications. Cells were washed with DPBS and lysed in 250 μl of TRIzol per 1 × 10^5^ to 1 × 10^7^ cells. Chloroform (200 μl per 1 ml of TRIzol) was added, and phase separation was performed by centrifugation (10,000 rpm, 30 min, 4°C). The aqueous phase was transferred to tubes containing 1 μl of glycogen blue (30 μg/ml), and RNA was precipitated with 0.5 volume of isopropanol at −80°C overnight. Pellets were recovered by centrifugation (10,000 rpm, 60 min, 4°C), washed twice with 75% ethanol, air-dried, and resuspended in 20 to 50 μl of nuclease-free water. RNA concentration and purity were assessed by NanoDrop spectrophotometry, and samples were stored at −80°C.

cDNA was synthesized from total RNA using the reagents and volumes listed in Table 2. Reaction mixtures were incubated at 25°C for 10 min, 42°C for 60 min, and 70°C for 10 min in a Bio-Rad T100 thermal cycler. cDNA was diluted with ribonuclease-free water and stored at −20°C.

### Quantitative PCR

qPCR was performed in 10-μl reactions containing 1 μl of cDNA, using the ViiA7 RUO Thermal Cycler (Thermo Fisher Scientific) with specific probes and primers (Table 2). Cycling conditions were initial denaturation at 95°C for 1 min, followed by 40 cycles of 95°C for 15 s and 60°C for 30 s. Samples were run in duplicate, and amplification plots were used to confirm quantification within the exponential phase. Cycle threshold values were averaged, with thresholds set within the linear lower third of each amplification curve. Target gene expression was normalized to the housekeeping genes GAPDH and SDHA, and presented as normalized RV copies.

### RVA1B infection of PCLSs

PCLSs were infected with RVA1B using a high virus titer of 5.5 × 10^7^ plaque-forming units/ml, corresponding to 1.65 × 10^7^ infectious units in the 300-μl inoculum applied per slice.

### Data quantification

Cellular events, including extrusions and total cell numbers, were quantified using NIS-Elements software (Nikon, v5.21.03). For time-lapse imaging, frames corresponding to 2-hour intervals were analyzed. Extruding cells were distinguished from dividing cells by the absence of daughter cell formation and by their floating morphology tethered to the monolayer. Cell counts were obtained using GA Analysis in NIS-Elements, with thresholds for brightness and size applied automatically and verified visually.

For fixed immunostained samples, quantification was performed by *Z*-stack inspection in NIS-Elements. Extruded cells were identified by actin rings at the basal plane and nuclei positioned above the monolayer. Nuclei were segmented automatically using GA Analysis, with segmentation accuracy confirmed visually. Images were further processed using Fiji (ImageJ).

### Image and statistical analysis

Image presentation and statistical analyses were performed in GraphPad Prism (v10.3.1). Data represent the means ± SEM from at least three independent biological replicates (*n* = 3). Statistical differences were evaluated using independent *t* tests or one-way analysis of variance (ANOVA) with Dunnett’s or Tukey’s post hoc tests, as specified in the figure legends.

## References

[1] S. E. Jacobs, D. M. Lamson, K. St. George, T. J. Walsh, Human rhinoviruses. Clin. Microbiol. Rev. 26, 135–162 (2013). 10.1128/CMR.00077-12 PMC3553670 23297263

[2] H. Ganjian, C. Rajput, M. Elzoheiry, U. Sajjan, Rhinovirus and innate immune function of airway epithelium. Front. Cell. Infect. Microbiol. 10, 277 (2020). 10.3389/fcimb.2020.00277 PMC7316886 32637363

[3] C. Esneau, N. Bartlett, Y. A. Bochkov, “Rhinovirus structure, replication, and classification,” in *Rhinovirus Infections* (Elsevier, 2019), pp. 1–23; https://linkinghub.elsevier.com/retrieve/pii/B9780128164174000019.

[4] S. Seo, A. Waghmare, E. M. Scott, H. Xie, J. M. Kuypers, R. C. Hackman, A. P. Campbell, S.-M. Choi, W. M. Leisenring, K. R. Jerome, J. A. Englund, M. Boeckh, Human rhinovirus detection in the lower respiratory tract of hematopoietic cell transplant recipients: Association with mortality. Haematologica 102, 1120–1130 (2017). 10.3324/haematol.2016.153767 PMC5451345 28183847

[5] D. Blaas, R. Fuchs, Mechanism of human rhinovirus infections. Mol. Cell. Pediatr. 3, 21 (2016). 10.1186/s40348-016-0049-3 PMC4889530 27251607

[6] S. Ohsawa, J. Vaughen, T. Igaki, Cell extrusion: A stress-responsive force for good or evil in epithelial homeostasis. Dev. Cell 44, 284–296 (2018). 10.1016/j.devcel.2018.01.009 PMC6207186 29408235

[7] J. Rosenblatt, M. C. Raff, L. P. Cramer, An epithelial cell destined for apoptosis signals its neighbors to extrude it by an actin- and myosin-dependent mechanism. Curr. Biol. 11, 1847–1857 (2001). 10.1016/s0960-9822(01)00587-5 11728307

[8] G. Slattum, Y. Gu, R. Sabbadini, J. Rosenblatt, Autophagy in oncogenic K-Ras promotes basal extrusion of epithelial cells by degrading S1P. Curr. Biol. 24, 19–28 (2014). 10.1016/j.cub.2013.11.029 PMC3932369 24361067

[9] Y. Gu, T. Forostyan, R. Sabbadini, J. Rosenblatt, Epithelial cell extrusion requires the sphingosine-1-phosphate receptor 2 pathway. J. Cell Biol. 193, 667–676 (2011). 10.1083/jcb.201010075 PMC3166871 21555463

[10] G. T. Eisenhoffer, P. D. Loftus, M. Yoshigi, H. Otsuna, C.-B. Chien, P. A. Morcos, J. Rosenblatt, Crowding induces live cell extrusion to maintain homeostatic cell numbers in epithelia. Nature 484, 546–549 (2012). 10.1038/nature10999 PMC4593481 22504183

[11] G. Slattum, K. M. McGee, J. Rosenblatt, P115 RhoGEF and microtubules decide the direction apoptotic cells extrude from an epithelium. J. Cell Biol. 186, 693–702 (2009). 10.1083/jcb.200903079 PMC2742179 19720875

[12] V. K. Dwivedi, C. Pardo-Pastor, R. Droste, J. N. Kong, N. Tucker, D. P. Denning, J. Rosenblatt, H. R. Horvitz, Replication stress promotes cell elimination by extrusion. Nature 593, 591–596 (2021). 10.1038/s41586-021-03526-y PMC8403516 33953402

[13] L. A. Knodler, B. A. Vallance, J. Celli, S. Winfree, B. Hansen, M. Montero, O. Steele-Mortimer, Dissemination of invasive *Salmonella* via bacterial-induced extrusion of mucosal epithelia. Proc. Natl. Acad. Sci. U.S.A. 107, 17733–17738 (2010). 10.1073/pnas.1006098107 PMC2955089 20876119

[14] L. A. Knodler, S. M. Crowley, H. P. Sham, H. Yang, M. Wrande, C. Ma, R. K. Ernst, O. Steele-Mortimer, J. Celli, B. A. Vallance, Noncanonical inflammasome activation of caspase-4/caspase-11 mediates epithelial defenses against enteric bacterial pathogens. Cell Host Microbe 16, 249–256 (2014). 10.1016/j.chom.2014.07.002 PMC4157630 25121752

[15] E. E. Bastounis, F. Serrano-Alcalde, P. Radhakrishnan, P. Engström, M. J. Gómez-Benito, M. S. Oswald, Y.-T. Yeh, J. G. Smith, M. D. Welch, J. M. García-Aznar, J. A. Theriot, Mechanical competition triggered by innate immune signaling drives the collective extrusion of bacterially infected epithelial cells. Dev. Cell 56, 443–460.e11 (2021). 10.1016/j.devcel.2021.01.012 PMC7982222 33621492

[16] S. M. Crowley, X. Han, J. M. Allaire, M. Stahl, I. Rauch, L. A. Knodler, B. A. Vallance, Intestinal restriction of *Salmonella* Typhimurium requires caspase-1 and caspase-11 epithelial intrinsic inflammasomes. PLOS Pathog. 16, e1008498 (2020). 10.1371/journal.ppat.1008498 PMC7179941 32282854

[17] P. A. Ngo, M. F. Neurath, R. López-Posadas, Impact of epithelial cell shedding on intestinal homeostasis. Int. J. Mol. Sci. 23, 4160 (2022). 10.3390/ijms23084160 PMC9027054 35456978

[18] N. Naseer, J. Zhang, R. Bauer, D. A. Constant, T. J. Nice, I. E. Brodsky, I. Rauch, S. Shin, *Salmonella enterica* serovar Typhimurium induces NAIP/NLRC4- and NLRP3/ASC-independent, caspase-4-dependent inflammasome activation in human intestinal epithelial cells. Infect. Immun. 90, e00663-21 (2022). 10.1128/iai.00663-21 PMC9302179 35678562

[19] Z. Zhang, J. Zou, Z. Shi, B. Zhang, L. Etienne-Mesmin, Y. Wang, X. Shi, F. Shao, B. Chassaing, A. T. Gewirtz, IL-22–induced cell extrusion and IL-18–induced cell death prevent and cure rotavirus infection. Sci. Immunol. 5, eabd2876 (2020). 10.1126/sciimmunol.abd2876 PMC7709835 33008915

[20] J. Moshiri, A. R. Craven, S. B. Mixon, M. R. Amieva, K. Kirkegaard, Mechanosensitive extrusion of Enterovirus A71-infected cells from colonic organoids. Nat. Microbiol. 8, 629–639 (2023). 10.1038/s41564-023-01339-5 PMC10066035 36914754

[21] Y. Li, S. Singh, H. L. Briggs, J. E. Kreger, A. L. Sliwicki, E. L. Eberhardt, S. Kuo, J. A. Czapla, J. K. Bentley, H. R. Flori, A. Horani, S. L. Brody, M. B. Hershenson, Rhinovirus C15 infection induces airway epithelial cell remodeling and robust inflammatory responses: Potential implications for airway obstruction in children. Mucosal Immunol. 18, 1341–1352 (2025). 10.1016/j.mucimm.2025.09.002 PMC12908968 40975321

[22] R. B. Turner, J. O. Hendley, J. M. Gwaltney, Shedding of infected ciliated epithelial cells in rhinovirus colds. J. Infect. Dis. 145, 849–853 (1982). 10.1093/infdis/145.6.849 6282984

[23] B. Hoorn, D. A. J. Tyrrell, A new virus cultivated only in organ cultures of human ciliated epithelium. Arch. Gesamte Virusforsch. 18, 210–225 (1966). 10.1007/BF01241842 4293705

[24] S. E. Reed, A. Boyde, Organ cultures of respiratory epithelium infected with rhinovirus or parainfluenza virus studied in a scanning electron microscope. Infect. Immun. 6, 68–76 (1972). 10.1128/iai.6.1.68-76.1972 PMC422492 4343933

[25] S. A. Gudipaty, J. Rosenblatt, Epithelial cell extrusion: Pathways and pathologies. Semin. Cell Dev. Biol. 67, 132–140 (2017). 10.1016/j.semcdb.2016.05.010 PMC5116298 27212253

[26] D. C. Bagley, T. Russell, E. Ortiz-Zapater, S. Stinson, K. Fox, P. F. Redd, M. Joseph, C. Deering-Rice, C. Reilly, M. Parsons, C. Brightling, J. Rosenblatt, Bronchoconstriction damages airway epithelia by crowding-induced excess cell extrusion. Science 384, 66–73 (2024). 10.1126/science.adk2758 PMC7619646 38574138

[27] R. R. Reddel, Y. Ke, B. I. Gerwin, M. G. McMenamin, J. F. Lechner, R. T. Su, D. E. Brash, J.-B. Park, J. S. Rhim, C. C. Harris, Transformation of human bronchial epithelial cells by infection with SV40 or adenovirus-12 SV40 hybrid virus, or transfection via strontium phosphate coprecipitation with a plasmid containing SV40 early region genes. Cancer Res. 48, 1904–1909 (1988). 2450641

[28] X. Han, T. Na, T. Wu, B.-Z. Yuan, Human lung epithelial BEAS-2B cells exhibit characteristics of mesenchymal stem cells. PLOS ONE 15, e0227174 (2020). 10.1371/journal.pone.0227174 PMC6941928 31900469

[29] D. R. Gude, S. E. Alvarez, S. W. Paugh, P. Mitra, J. Yu, R. Griffiths, S. E. Barbour, S. Milstien, S. Spiegel, Apoptosis induces expression of sphingosine kinase 1 to release sphingosine-1-phosphate as a “come-and-get-me” signal. FASEB J. 22, 2629–2638 (2008). 10.1096/fj.08-107169 PMC2493451 18362204

[30] D. Andrade, J. Rosenblatt, Apoptotic regulation of epithelial cellular extrusion. Apoptosis 16, 491–501 (2011). 10.1007/s10495-011-0587-z PMC4393655 21399977

[31] L. Deszcz, E. Gaudernak, E. Kuechler, J. Seipelt, Apoptotic events induced by human rhinovirus infection. J. Gen. Virol. 86, 1379–1389 (2005). 10.1099/vir.0.80754-0 15831950

[32] E. A. Slee, H. Zhu, S. C. Chow, M. MacFARLANE, D. W. Nicholson, G. M. Cohen, Benzyloxycarbonyl-Val-Ala-Asp (OMe) fluoromethylketone (Z-VAD.FMK) inhibits apoptosis by blocking the processing of CPP32. Biochem. J. 315, 21–24 (1996). 10.1042/bj3150021 PMC1217173 8670109

[33] J. J. Malin, I. Suárez, V. Priesner, G. Fätkenheuer, J. Rybniker, Remdesivir against COVID-19 and other viral diseases. Clin. Microbiol. Rev. 34, e00162-20 (2020). 10.1128/CMR.00162-20 PMC7566896 33055231

[34] A. Simonis, S. J. Theobald, G. Fätkenheuer, J. Rybniker, J. J. Malin, A comparative analysis of remdesivir and other repurposed antivirals against SARS-CoV-2. EMBO Mol. Med. 13, e13105 (2021). 10.15252/emmm.202013105 PMC7646058 33015938

[35] C. Bae, F. Sachs, P. A. Gottlieb, The mechanosensitive ion channel Piezo1 is inhibited by the peptide GsMTx4. Biochemistry 50, 6295–6300 (2011). 10.1021/bi200770q PMC3169095 21696149

[36] T. M. Suchyna, J. H. Johnson, K. Hamer, J. F. Leykam, D. A. Gage, H. F. Clemo, C. M. Baumgarten, F. Sachs, Identification of a peptide toxin from *Grammostola spatulata* spider venom that blocks cation-selective stretch-activated channels. J. Gen. Physiol. 115, 583–598 (2000). 10.1085/jgp.115.5.583 PMC2217226 10779316

[37] R. Fuchs, D. Blaas, Productive entry pathways of human rhinoviruses. Adv. Virol. 2012, 750130 (2012). 10.1155/2012/826301 PMC3513715 23227049

[38] T. A. Hill, C. P. Gordon, A. B. McGeachie, B. Venn-Brown, L. R. Odell, N. Chau, A. Quan, A. Mariana, J. A. Sakoff, M. Chircop, P. J. Robinson, A. McCluskey, Inhibition of dynamin mediated endocytosis by the *dynoles*—Synthesis and functional activity of a family of indoles. J. Med. Chem. 52, 3762–3773 (2009). 10.1021/jm900036m 19459681

[39] C. Gundu, V. K. Arruri, P. Yadav, U. Navik, A. Kumar, V. S. Amalkar, A. Vikram, R. R. Gaddam, Dynamin-independent mechanisms of endocytosis and receptor trafficking. Cells 11, 2557 (2022). 10.3390/cells11162557 PMC9406725 36010634

[40] R. M. Liesman, U. J. Buchholz, C. L. Luongo, L. Yang, A. D. Proia, J. P. DeVincenzo, P. L. Collins, R. J. Pickles, RSV-encoded NS2 promotes epithelial cell shedding and distal airway obstruction. J. Clin. Invest. 124, 2219–2233 (2014). 10.1172/JCI72948 PMC4001550 24713657

[41] W.-H. W. Lin, A. J. Tsay, E. N. Lalime, A. Pekosz, D. E. Griffin, Primary differentiated respiratory epithelial cells respond to apical measles virus infection by shedding multinucleated giant cells. Proc. Natl. Acad. Sci. U.S.A. 118, e2013264118 (2021). 10.1073/pnas.2013264118 PMC7980467 33836570

[42] C. E. Hippee, L. A. Durnell, J. W. Kaufman, E. Murray, B. K. Singh, P. L. Sinn, Epithelial-to-mesenchymal transition and live cell extrusion contribute to measles virus release from human airway epithelia. J. Virol. 99, e01220–e01224 (2025). 10.1128/jvi.01220-24 PMC11852777 39791903

[43] S. M. Warner, S. Wiehler, A. N. Michi, D. Proud, Rhinovirus replication and innate immunity in highly differentiated human airway epithelial cells. Respir. Res. 20, 150 (2019). 10.1186/s12931-019-1120-0 PMC6626354 31299975

[44] N. Lopez-Souza, G. Dolganov, R. Dubin, L. A. Sachs, L. Sassina, H. Sporer, S. Yagi, D. Schnurr, H. A. Boushey, J. H. Widdicombe, Resistance of differentiated human airway epithelium to infection by rhinovirus. Am. J. Physiol. Lung Cell. Mol. Physiol. 286, L373–L381 (2004). 10.1152/ajplung.00300.2003 14711802

[45] A. Fortunato, A. Fleming, A. Aktipis, C. C. Maley, Upregulation of DNA repair genes and cell extrusion underpin the remarkable radiation resistance of *Trichoplax adhaerens*. PLoS Biol. 19, e3001471 (2021). 10.1371/journal.pbio.3001471 PMC8635375 34788294

[46] A. Grata, R. Levayer, Epithelial cell extrusion at a glance. J. Cell Sci. 138, jcs263786 (2025). 10.1242/jcs.263786 40270445

[47] I. Fournon-Berodia, N. Bruderer, L. Christiaen, P. R. H. Steinmetz, Epithelial cell extrusion underlies starvation-induced cell loss in a sea anemone. Nat. Commun. 17, 7681 (2026). 10.1038/s41467-026-74437-7 PMC13433931 42310303

[48] A. Papi, S. L. Johnston, Rhinovirus infection induces expression of its own receptor intercellular adhesion molecule 1 (ICAM-1) via increased NF-κB-mediated transcription. J. Biol. Chem. 274, 9707–9720 (1999). 10.1074/jbc.274.14.9707 10092659

[49] C. Lei, J. Yang, J. Hu, X. Sun, On the calculation of TCID_50_ for quantitation of virus infectivity. Virol. Sin. 36, 141–144 (2021). 10.1007/s12250-020-00230-5 PMC7973348 32458296

