# Supplementary material for: Airway epithelia clear rhinovirus infection via two waves of cell extrusion
Source: Sci Adv. 2026 Sep 25;12(39):eaef2562. doi: 10.1126/sciadv.aef2562 (PMC13614396; doi:10.1126/sciadv.aef2562)
Supplement: Supplementary file 1 [file sciadv.aef2562_sm.pdf]

Science Advances
AAAS

Supplementary Materials for

Airway epithelia clear rhinovirus infection via two waves of cell extrusion

Faith Fore *et al.*

Corresponding author: Jody Rosenblatt, jody.rosenblatt@kcl.ac.uk

Sci. Adv. **12**, eaef2562 (2026)
DOI: 10.1126/sciadv.aef2562

The PDF file includes:

Figs. S1 to S3
Legends for movies S1 to S4

Other Supplementary Material for this manuscript includes the following:

Movies S1 to S4

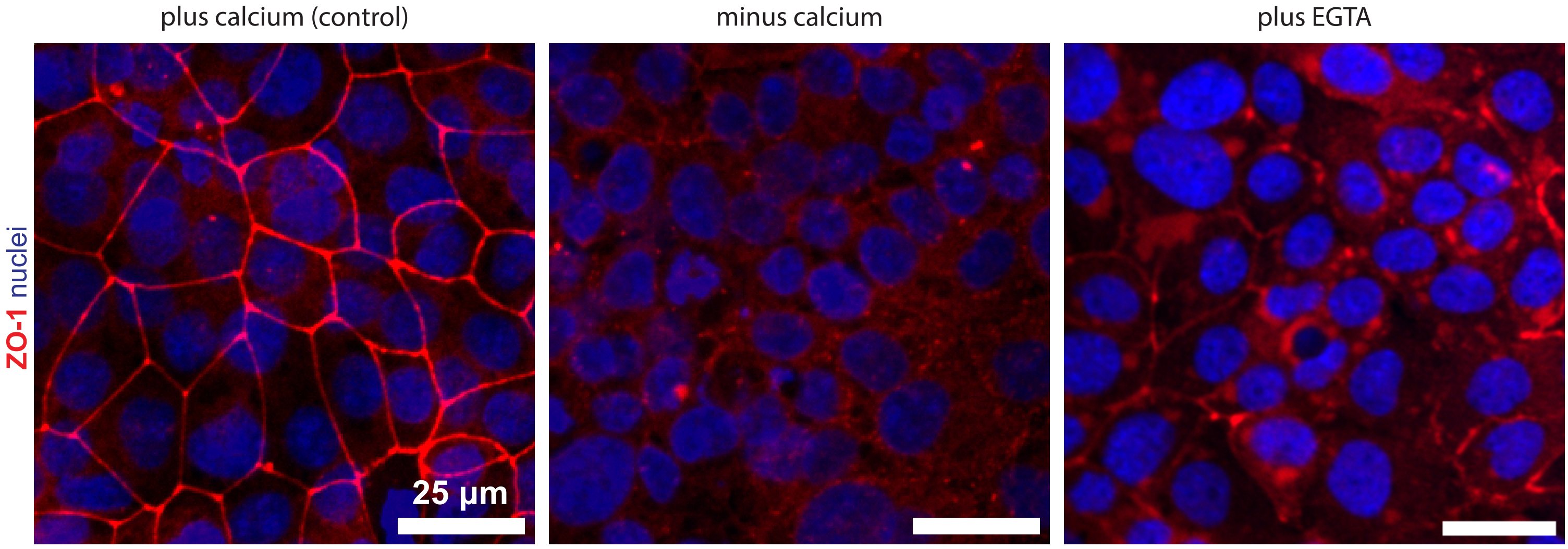

Fig. S1: Disruption of tight junctions in 16HBE14o⁻ monolayers. Representative confocal images showing disruption of tight junctions in 16HBE14o⁻ monolayers by incubation in calcium-free medium or by chelating extracellular calcium with EGTA. Tight junctions were visualised using ZO-1 (zonula occludens-1) staining and nuclei with DAPI. Scale bar, 25 µm

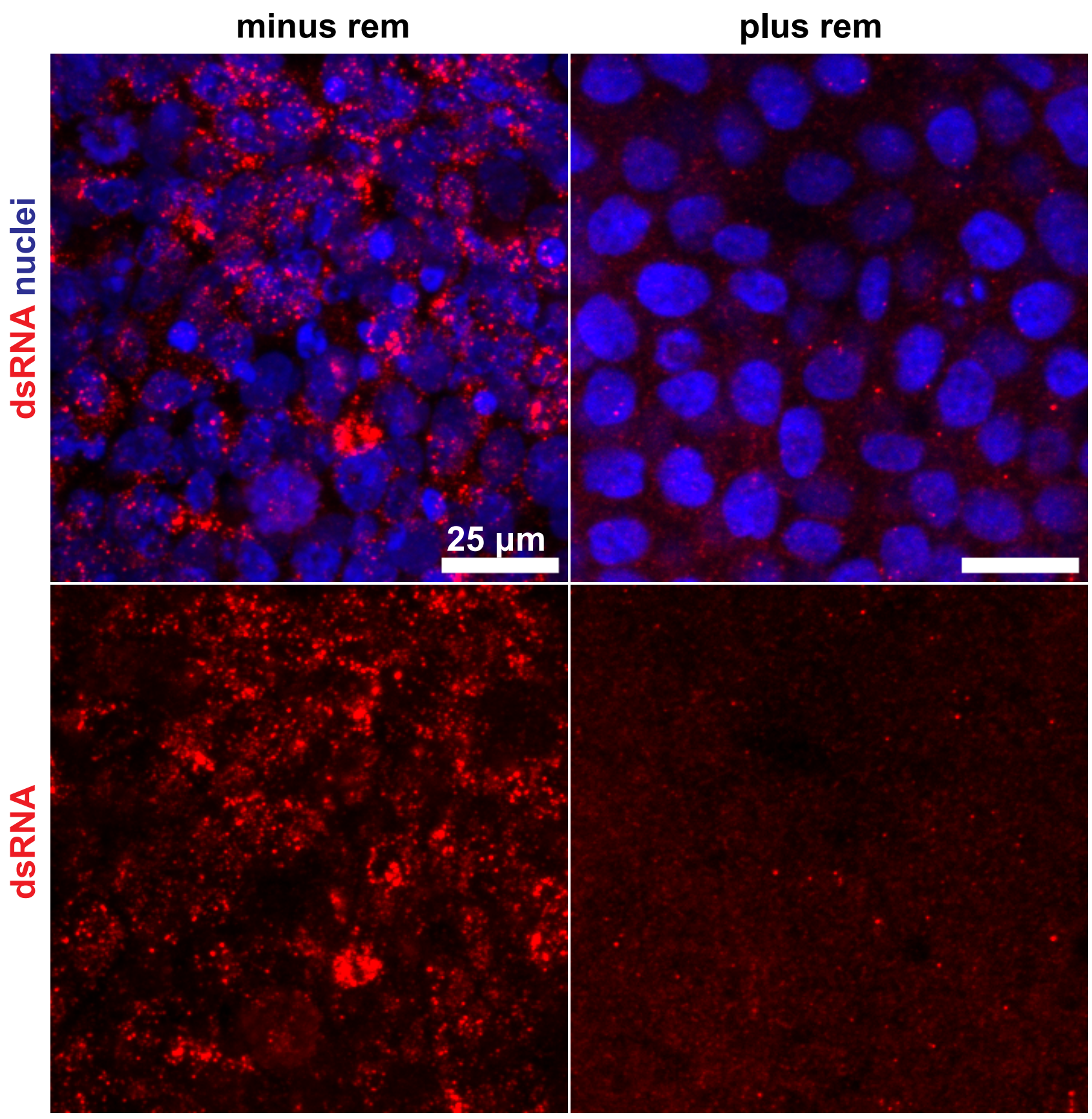

Fig. S2: Remdesivir inhibits replication of RV-A16 in HeLa-H1 cells. Representative confocal images of HeLa-H1 cells infected with RV-A16 for 24 hours ± remdesivir Viral replication was visualised by dsRNA staining and nuclei with DAPI (blue). Scale bar, 25 µm.

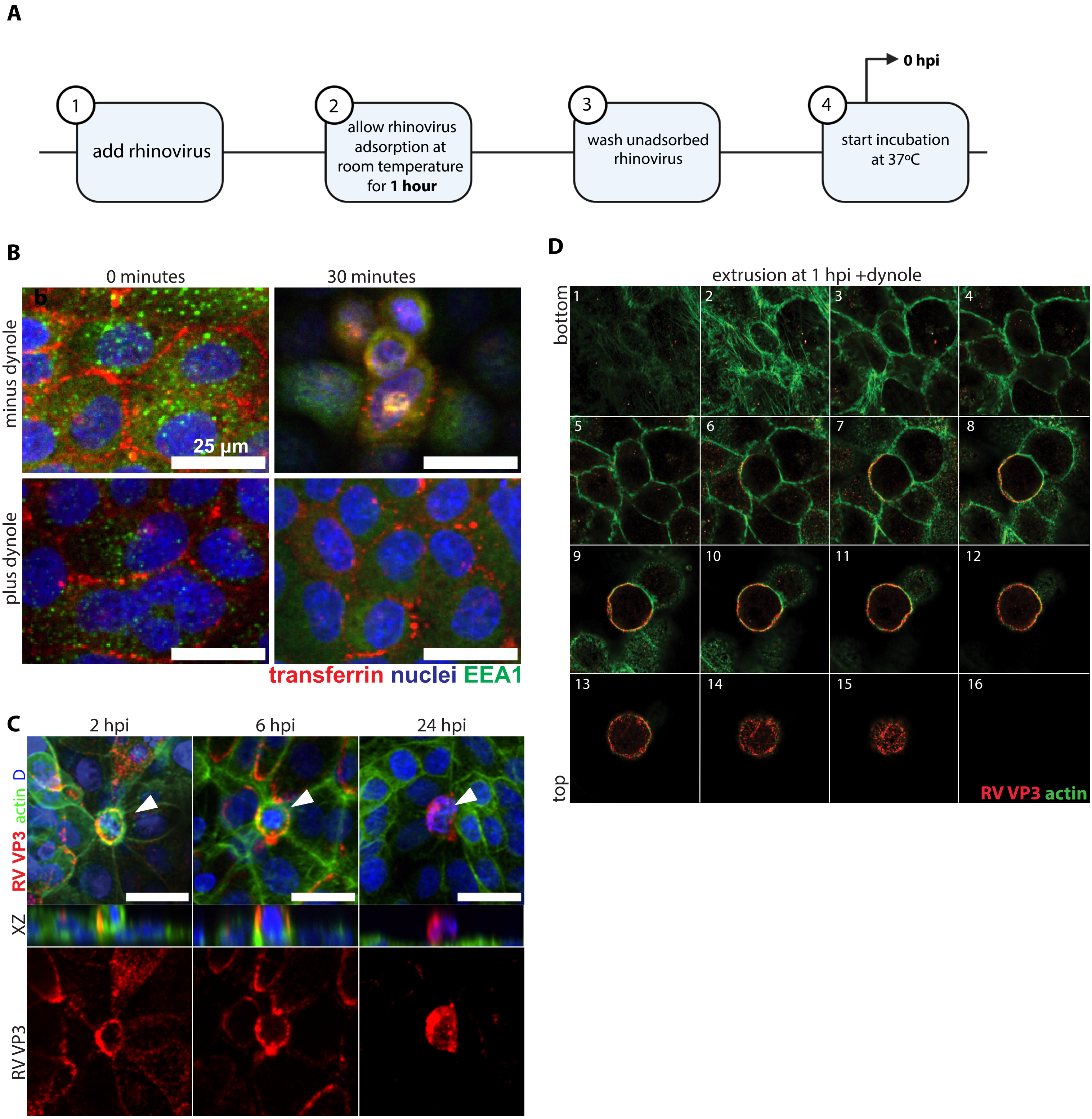

Fig. S3: The early VICE wave is initiated during rhinovirus adsorption. A, Schematic of the infection experiment, indicating adsorption and infection steps (0 hpi). **B,** Representative confocal images showing transferrin uptake inhibited by Dynole 34-2, where early endosomes were also immunostained. **C,** Scanning confocal z-stack from basal to apical planes of a VICE cell at 0 hpi (one hour of RV-A16 adsorption at room temperature). **D,** Representative confocal images of RV-A16-infected cells +Dynole 34-2. Scale bar, 25 µm.

Supplementary movies

SMovie1: Time-lapse imaging of mock-infected 16HBE14o$^-$ monolayers.

Representative time-lapse sequence of mock-infected 16HBE14o$^-$ monolayers imaged for 12 hours showing few extrusion events. Green arrows indicate extrusion events, and red circles indicate representative dividing cells. Scale bar, 25 µm.

SMovie2: Time-lapse imaging of RV-A16-infected 16HBE14o$^-$ monolayers.

Representative time-lapse sequence of RV-A16-infected 16HBE14o$^-$ monolayers imaged for 12 hours, showing frequent extrusion events. Green arrows indicate extrusion events, and red circles indicate representative dividing cells. Scale bar, 25 µm.

SMovie3: Time-lapse imaging of RV-A16-infected 16HBE14o$^-$ monolayers treated with JTE-013.

Representative time-lapse sequence of RV-A16-infected 16HBE14o$^-$ monolayers treated with the $S1P_2$ receptor antagonist JTE-013, imaged for 12 hours showing extrusion events relative to untreated infected monolayers. Green arrows indicate extrusion events, and red circles indicate representative dividing cells. Scale bar, 25 µm.

SMovie4: Extruded ciliated epithelial cells remain viable following RV-A16 infection.

Live imaging of apical washes collected from fully differentiated primary human airway epithelial cultures following RV-A16 infection. Beating cilia demonstrate that extruded epithelial cells remain viable following extrusion. Scale bar, 25 µm.
